# Simultaneous Quantitation and Validation of Triterpenoids and Phytosteroids in *Phaseolus angularis* Seeds

**DOI:** 10.3390/molecules190710309

**Published:** 2014-07-16

**Authors:** Joo Tae Hwang, Hyun-Mee Oh, Mi-Hwa Kim, Hyung Jae Jeong, Chul Lee, Hyun-Jae Jang, Seung Woong Lee, Chan Sun Park, Mun-Chual Rho

**Affiliations:** Eco-friendly Material Research Center, Korea Research Institute of Bioscience and Biotechnology, Jeongeup-si 580-185, Korea

**Keywords:** *Phaseolus angularis* seeds, TLR4, HPLC-UV, validation

## Abstract

A reproducible analytical method using reverse-phase high liquid performance chromatography combined with UV detecting was developed for the quantitative determination of four compounds isolated from the ethanol extract of *Phaseolus angularis* seeds (PASE): oleanolic acid (**1**), oleanolic acid acetate (**2**), stigmasterol (**3**) and β-sitosterol (**4**). This method was fully validated in terms of linearity (r^2^ > 0.999), accuracy (98.5%–100.8%), precision (<0.92%), LOD (<0.0035 mg/mL), and LOQ (<0.0115 mg/mL). The effects of the PASE and isolated compounds **1**–**4** on TLR4 activation were tested in THP1-Blue cells. Among the tested substances, compound **2** showed potent inhibitory activity with an IC_50_ value of 3.89 ± 0.17 µM.

## 1. Introduction

*Phaseolus angularis* seeds (PAS) as a very important crop in East Asia that has mainly been cultivated in Korea, Japan, northern and central China for many centuries. They are traditionally used as diuretic, antidote and a remedy for dropsy and beriberi [1]. Previous phytochemical and biological investigations on PAS have been reported that vignosides A and B, sesquiterpene glucosides isolated from a hot-water extract, inhibited the growth of human stomach cancer KATO III cells [2], vitexin and isovitexin, flavonoids from the EtOAc-soluble fraction, showed inhibitory activities against α-glucosidase [3], and catechin analogues from a 40% ethanol extract showed anti-tumor, antioxidative and anti-diabetic activities [4]. Especially, the 80% ethanol extract, containing polyphenolic compounds, had an effect on the systolic blood pressure and macrophage infiltration in the heart and kidney of spontaneously hypertensive rats [5]. Recently, it was also reported that PAS ethanol extract (PASE) showed potent inhibitory effects on IL-6/STAT3 and toll-like receptors (TLRs) signaling [6,7,8]. Taken together, the evidence suggests great interest in PAS for its phytochemicals and biological activities.

**Figure 1:**
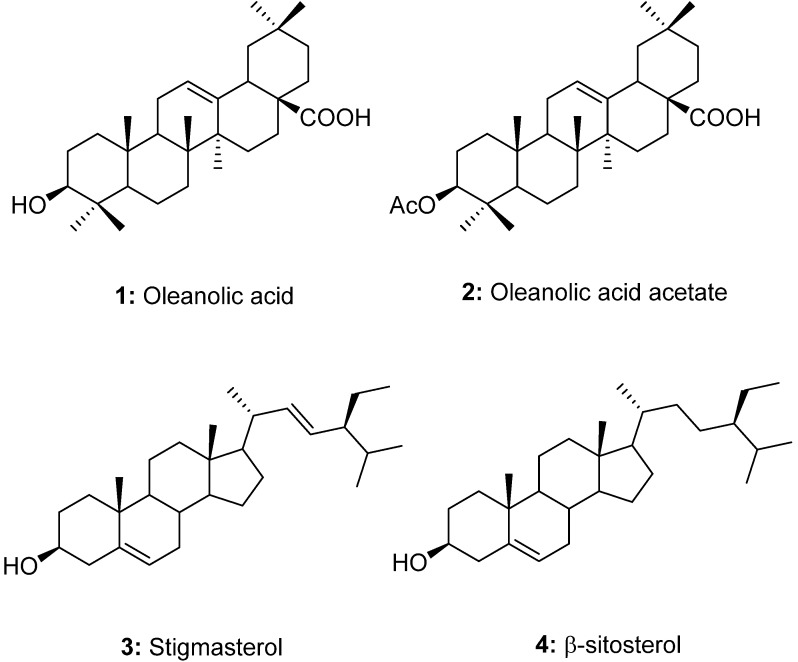
Chemical structures of isolated compounds **1**–**4**.

A number of studies have suggested that TLRs play a critical role in the innate immune response. Among TLRs, TLR4 is activated by lipopolysaccharides (LPS) and serves as the primary mediator of the pathogenesis of inflammation [9]. Previously, the inhibitory effect of PASE on the TLR4 activation and TLR4-mediated inflammatory response was reported [10]. In this study, we isolated four constituents from PASE to identify the active compounds that are responsible for the inhibitory effect on TLR4 activity. To clarify the crucial phytochemicals of PASE, the reproducible analytical method was also developed for the quantitative determination of four compounds as well as the full validation of the developed method including linearity, accuracy, precision, LOD and LOQ. Although, the analytical method of isolated compounds were investigated, it was, to the best of our knowledge, the first attempt to develop the simultaneous determination of compounds **1**–**4** (Figure 1) in PAS [11,12]. This paper thus describes the quantification and validation of isolated compounds with TLR4 inhibitory effects from PASE.

## 2. Results and Discussion

### 2.1. Optimization of Chromatographic Conditions

**Figure 2:**
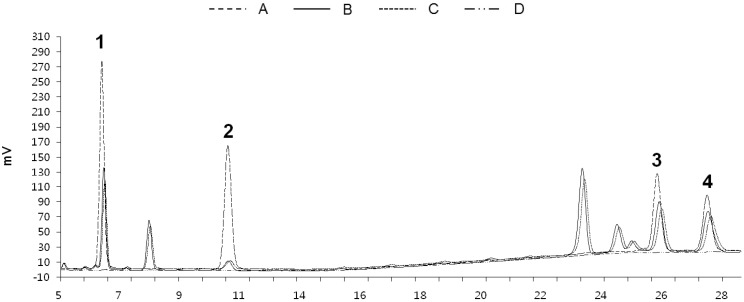
HPLC chromatograms (210 nm) of the standard compounds of PASE (**A**, 3 mg/mL); the ethanol extract of PAS (**B**); the ethyl acetate extract of PAS (**C**); and the water extract of PAS (**D**). Peak numbers refer to the compounds shown in Table 1.

Various analytical columns, mobile phases, and elution systems employing the mixture of isolated compounds were tested for the determination of the optimal condition. C_18_ columns are more popular than C_8_ columns in HPLC analysis, but were found to be difficult to use in this case due to the long retention time shifts and peak broadening, so a Luna-C_8_ column was employed. To select the optimum mobile phase for baseline separation, different solvent mixture components were tested, *i*.*e*., methanol, acetonitrile, and water. The four compounds could be eluted efficiently and simultaneously by a mixture of methanol (A) and water (B). Also, addition of 0.1% phosphoric acid to water provided improved peak shapes. In terms of gradient elution system and column temperature, the gradient elution of A/B = 85/15 (0–10 min) → A/B = 90/10 (20–30 min) with flow rate 1 mL/min and 40 °C showed the optimal separation performance. The wavelength for the detection was set at 210 nm, where all compounds exhibited the maximum absorption. Under these HPLC conditions, all compounds were free of interference with any other ingredients and showed retention times of 6.53 (**1**), 10.92 (**2**), 26.09 (**3**), and 27.82 (**4**) min, respectively (Figure 2).

**Table 1:** Content of compounds in the PAS (mg/g).

Compound	Contents (*n* = 3)		
	EtOH	Ethyl Acetate	Water
**1**	3.76 ± 0.04 *^a^*	2.83 ± 0.05	0.04 ± 0.20
**2**	0.72 ± 0.10	0.48 ± 0.49	0.02 ± 1.18
**3**	5.76 ± 0.05	4.07 ± 0.25	0.03 ± 1.35
**4**	7.38 ± 0.07	5.25 ± 0.17	0.19 ± 0.98

*^a^* Standard error (mg/g).

### 2.2. Validation of Developed Analytical Method

#### 2.2.1. Linearity

Linearity studies were performed to ensure whether the developed analytical methods could estimate different concentration of standards. Ten total concentrations, *i*.*e*., 0.005, 0.01, 0.03, 0.05, 0.1, 0.3, 0.5, 1, 2, and 3 mg/mL, were analyzed with three repetitions for each compound,. As shown in Table 2, the individual calibration curves exhibited good linearity for all components, and the *r*^2^ values were more than 0.999.

**Table 2:** Linearity, LOD and LOQ of the standard compounds (**1**–**4**).

Compounds	t*_R_* (min)	Equation(Linear Model) *^a^*	Linear Range (mg/mL)	*r* ^2^ *^b^*	LOD *^c^* (mg/mL)	LOQ *^d^* (mg/mL)
**1**	6.53	y = 1,048,348x − 3930	0.005–3	0.9993	0.0016	0.0052
**2**	10.92	y = 958,420x − 3329	0.005–3	0.9993	0.0003	0.0011
**3**	26.09	y = 626,343x − 578	0.005–3	0.9990	0.0023	0.0076
**4**	27.82	y = 509,396x − 2377	0.005–3	0.9993	0.0035	0.0115

*^a^* y: peak area at 210 nm; x: standard concentration (mg/mL). *^b^* *r*^2^: coefficient of determination with 10 indicated points in the calibration curves. *^c^* LOD: limit of detection, S/N = 3 (*n* = 6). *^d^* LOQ: limit of quantification, S/N = 10 (*n* = 6).

#### 2.2.2. Precision and Accuracy

In the intra-day variability test, the RSD values of compounds were measured within the range of 0.01%–0.08% for **1**, 0.49%–0.83% for **2**, 0.25%–0.69% for **3**, and 0.10%–0.92% for **4**, respectively. The RSD values on three consecutive days were 0.65%–0.90% for **1**, 0.52%–0.63% for **2**, 0.75%–0.89% for **3**, and 0.56%–0.87% for **4**, respectively. In terms of the accuracy, it was measured as 98.5%–99.8% for **1**, 100.1–100.8 for **2**, 99.4%–100.8% for **3**, and 99.2%–100.7% for **4**, respectively. These results (Table 3) indicate that the precision and accuracy of the assays are reliable and precise for the simultaneous analysis of four compounds in the PAS.

**Table 3:** Accuracy, intra- and inter-day precision of the standard compounds **1**–**4**.

Compounds	Spiked Amount (mg/mL)	Content (mg/mL)		Recovery Test (%, *n* = 3)	Precision Test (*n* = 3)	
		Expected	Measured		Intra-Day RSD *^a^* (%)	Inter-Day RSD (%)
**1**	0.10	3.8593	3.8521	99.8	0.04	0.85
	0.30	4.0593	3.9991	98.5	0.08	0.65
	1.00	4.7593	4.7506	99.8	0.01	0.90
**2**	0.02	0.7390	0.7446	100.8	0.62	0.63
	0.06	0.7790	0.7805	100.2	0.83	0.52
	0.20	0.9109	0.9203	100.1	0.49	0.89
**3**	0.15	5.9080	5.9644	100.8	0.69	0.78
	0.45	6.2090	6.1723	99.4	0.25	0.89
	1.50	7.2590	7.3032	100.6	0.36	0.75
**4**	0.20	7.5800	7.6294	100.7	0.92	0.56
	0.60	7.9800	7.9151	99.2	0.66	0.86
	2.00	9.3800	9.3890	100.0	0.10	0.87

*^a^* RSD: relative standard deviation.

#### 2.2.3. LOD, and LOQ

Limit of detection (LOD) and limit of quantification (LOQ) values were calculated from signal-to-noise ratio (S/N) method. LODs and LOQs values were less calculated than 0.0035 and 0.0115 mg/mL, indicating that LODs and LOQs values were lower than contents of compounds in the PAS.

### 2.3. Quantitation of Compounds **1**–**4**

The developed and validated analytical method was used to quantify oleanolic acid (**1**), oleanolic acid acetate (**2**), stigmasterol (**3**) and β-sitosterol (**4**) in the PAS, and the contents of the four compounds were successfully determined in three extraction solvents including water, ethyl acetate and ethanol (Table 1). As a result, it was shown that the ethanol extract of PAS (PASE) had the highest contents of all compounds, while the content of **4** was the highest among the four compounds.

### 2.4. TLR4 Inhibitory Activity

**Figure 3:**
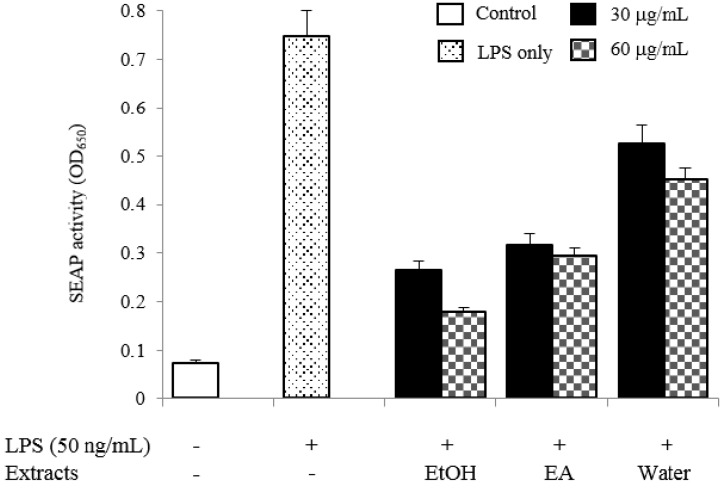
Inhibitory effects of PAS extracts on the SEAP activity.

**Figure 4:**
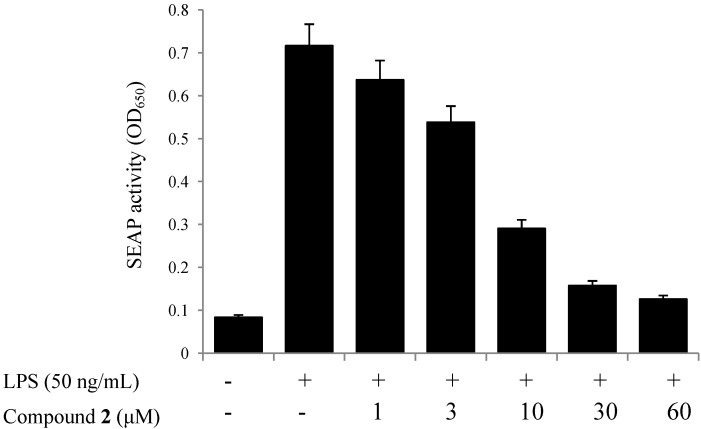
Inhibitory effects of compound **2** on the SEAP activity with indicated concentrations.

**Figure 5:**
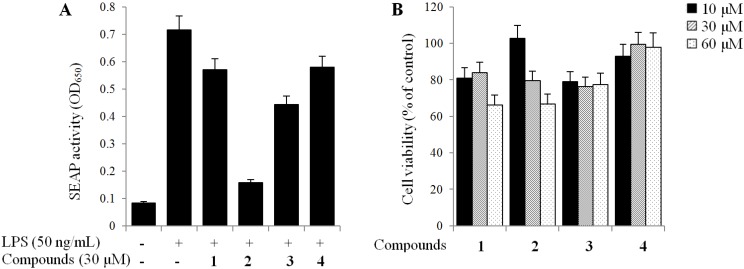
Inhibitory effects of compounds on the SEAP activity (**A**) and their cell viabilities (**B**).

We have assessed the TLR4 inhibitors from edible herbs. Among our candidates, we found that the ethanol extract of PAS showed strong TLR4 inhibitory activity in human monocyte THP1-Blue cells (IC_50_ < 30 µg/mL). As shown in Figure 3, all extracts exhibited significant inhibitory activities on TLR4 activation, especially ethanol extract (showed 71.6% inhibition at 30 µg/mL). Four isolated compounds (**1**–**4**) were also evaluated for their inhibitory effects on the SEAP activity to identify the active compounds that were responsible for its effect. As a result, oleanolic acid acetate (**2**) showed the most potent inhibitory effect with an IC_50_ value of 3.89 ± 0.17 µM (Figure 4). No cytotoxic activities were observed in the used concentrations, indicating that the effect of **2** was not related to any cytotoxicity (Figure 5).

## 3. Experimental

### 3.1. General Procedures

Analytical grade acetonitrile, methanol and water were purchased from J. T. Baker (Phillipsburg, NJ, USA). Extra pure grade ethyl alcohol, ethyl acetate and n-hexane were purchased from Dae-Jung Chemicals and Metals (Gyeonggi-do, Korea). NMR spectra were recorded on a Jeol-ECS 400 MHz NMR (Jeol, Tokyo, Japan) spectrometer using CDCl_3_ and pyridine-d_5_ as solvent and TMS was used as internal standard. Chemical shifts are presented in ppm. TLC analysis was performed on a pre-coated silica gel 60 F_254_ (0.24 mm, Merck, Whitehouse Station, NJ, USA). Open column chromatography was performed using a silica gel (Kieselgel 60, 70–230 mesh, Merck). Semi-preparative HPLC was performed on a Shimadzu HPLC system equipped with a quaternary pump (LC-20AD), auto-sampler (SIL-20A), UV-detector (SPD-20A), column oven (CTO-20A) and LC solution system (Shimadzu, Kyoto, Japan). The ESI and EI-MS spectra were acquired using a Shimadzu LC/MS 2020 system and GC/MS QP2010S system, respectively.

### 3.2. Materials and Chemicals

#### Plant Materials

*Phaseolus angularis* seeds were purchased from an herbal medicine store in Jeongeup-si, Jeonbuk, Korea. The authenticity of this plant was confirmed by Prof. Y.H. Kim at the College of Pharmacy of Chungnam National University. A voucher specimen was deposited at the Jeongeup Branch of KRIBB.

### 3.3. Isolation and Identification of Compounds **1**–**4**

Dried PAS (10 kg) was pulverized to fine powder and extracted twice with 95% ethanol (60 L) at 70 °C. After filtering, it was evaporated in a rotary evaporator to yield the ethanol extract (1.1 kg). Some of the ethanol extract (450 g) was suspended in distilled water and progressively partitioned with *n*-hexane, EtOAc and water. The *n*-hexane soluble fraction (80 g) was chromatographed on silica gel with a step gradient composed of *n*-hexane and EtOAc (100:0, 80:1, 50:1, 30:1, 15:1, 5:1, 2:1, 1:1, step gradient) to obtain twenty six bottles. These were combined into nine sub-fractions (A-I) by TLC profiles that were carried out on pre-coated silica gel (mobile phase: *n*-hexane/EtOAc). The recrystallization of sub-fraction C in methanol gave compounds **1** (40 mg) and **2** (12 mg). Compounds **3** (90 mg) and **4** (75 mg) were isolated from sub-fraction E by preparative HPLC (acetonitrile and water, 80/20, *v/v*). Chemical structures of isolates were determined by the comparison of spectroscopic data including ^1^H, ^13^C-NMR, ESI- and EI-MS with previously in the literature published values and were identified as oleanolic acid (**1**) [13], oleanolic acid acetate (**2**) [14], stigmasterol (**3**) [15] and β-sitosterol (**4**) [16].

#### 3.3.1. Oleanolic Acid (**1**)

White powder; ESI-MS *m*/*z* 455 [M−H]^−^; ^1^H-NMR (pyridine-*d_5_*) δ_H_ 0.75 (3H, s, Me-26), 0.87 (3H, s, Me-24), 0.89 (3H, s, Me-23), 0.91 (3H, s, Me-25), 0.96 (3H, s, Me-30), 1.12 (3H, s, Me-27), 2.87 (1H, m, H-18), 3.46 (1H, m, H-3), 5.52 (1H, m, H-12); ^13^C-NMR (pyridine-*d_5_*) δ_C_ 180.0 (C-28), 144.8 (C-13), 122.6 (C-12), 18.2 (C-3), 55.9 (C-5), 48.2 (C-9), 46.7 (C-8 and C-17), 46.6 (C-14), 39.8 (C-4), 39.0 (C-1), 37.4 (C-10), 33.4 (C-7), 33.4 (C-29), 34.3 (C-21), 31.0 (C-20), 28.2 (C-23), 28.1 (C-2), 28.1 (C-15), 23.8 (C-30), 23.8 (C-11), 23.8 (C-16), 18.8 (C-6), 17.5 (C-26), 16.5 (C-24), 15.6 (C-25).

#### 3.3.2. Oleanolic Acid Acetate (**2**)

White powder; ESI-MS *m/z* 497 [M−H]^−^; ^1^H-NMR (CDCl_3_) δ_H_ 0.67 (3H, s, Me-26), 0.79 (3H, s, Me-29), 0.87 (3H, s, Me-24), 0.88 (3H, s, Me-23), 1.00 (3H, s, Me-30), 1.05 (3H, s, Me-25), 1.18 (3H, s, Me-27), 2.06 (3H, s, OAc), 2.78 (1H, m, H-18), 4.42 (1H, m, H-3), 5.19 (1H, m, H-12); ^13^C-NMR (CDCl_3_) δ_C_ 178.6 (C-28), 171.0 (CH_3_COO), 143.6 (C-13), 122.5 (C-12), 80.9 (C-3), 55.3 (C-5), 48.0 (C-9), 46.6 (C-17), 41.5 (C-14), 39.0 (C-4), 38.0 (C-1), 37.7 (C-10), 33.8 (C-21), 33.1 (C-29), 32.8 (C-7), 30.7 (C-20), 29.7 (C-23), 28.0 (C-15), 27.7 (C-2), 24.0 (C-11), 23.6 (C-30), 23.4 (C-16), 22.8 (CH_3_COO), 18.1 (C-6), 17.2 (C-26), 16.7 (C-24), 15.5 (C-25).

#### 3.3.3. Stigmasterol (**3**)

White amorphous powder; EI-MS *m*/*z*; 413 [M−H]^−^; ^1^H-NMR (CDCl_3_) δ_H_ 0.71, 0.80, 0.82, 0.83, 0.91, 1.03, (each 3H, s, CH_3_ × 6), 3.51 (1H, tdd, 4.5, 4.2, 3.8 Hz, H-3), 4.98 (1H, m, H-20), 5.31 (1H, t, 6.1 Hz, H-5), 5.14 (1H, m, H-21); ^13^C-NMR (CDCl_3_) δ_C_ 141.1 (C-5), 138.7 (C-20), 129.6 (C-21), 121.8 (C-6), 72.1 (C-3), 56.8 (C-14), 56.2 (C-17), 50.2 (C-9), 46.1 (C-22), 42.4 (C-4), 42.4 (C-13), 40.6 (C-18), 39.9 (C-12), 37.6 (C-1), 36.6 (C-10), 32.1 (C-2), 31.8 (C-7), 31.8 (C-8), 29.6 (C-25), 29.3 (C-16), 25.4 (C-23), 24.4 (C-15), 21.7 (C-19), 21.5 (C-11), 20.2 (C-26), 19.8 (C-27), 18.9 (C-28), 12.2 (C-29), 12.1 (C-24).

#### 3.3.4. β-Sitosterol (**4**)

White amorphous powder; EI-MS *m*/*z*; 415 [M−H]^−^. ^1^H-NMR (CDCl_3_): 0.68, 0.81, 0.83, 0.84, 0.93, 1.01 (each 3H, s, CH_3_ × 6), 3.53 (1H, tdd, 4.5, 4.2, 3.8 Hz, H-3), 5.36 (1H, t, 6.4 Hz, H-5); ^13^C-NMR (CDCl_3_) δ_C_ 140.9 (C-5), 121.9 (C-6), 72.0 (C-3), 56.9 (C-14), 56.3 (C-17), 50.3 (C-9), 46.1 (C-22), 42.6 (C-13), 42.5 (C-4), 39.9 (C-12), 37.5 (C-1), 36.7 (C-10), 36.3 (C-18), 34.2 (C-20), 32.1 (C-7), 32.1 (C-8), 31.9 (C-2), 29.4 (C-25), 28.5 (C-16), 26.3 (C-15), 26.3 (C-21), 23.3 (C-23), 21.3 (C-11), 20.1 (C-26), 19.6 (C-27), 19.2 (C-19), 19.0 (C-28), 12.2 (C-24), 12.0 (C-29).

### 3.4. Sample Preparation for HPLC

The pulverized seeds of *Phaseolus angularis* (5.0 g) were transferred into a 100 mL flask and extracted into 35 mL of EtOH, EtOAc, and water at 70 °C for 4 h, respectively. After centrifuging for 10 min at 4,000 g, the supernatant was filtered with a filter paper (Advantec, Tokyo, Japan) and then evaporated to dryness using a rotary evaporator at reduced pressure. The residue was dissolved in CHCl_3_/MeOH (7/3, *v/v*, 2 mL) and then filtered again using a disposable syringe filter unit (0.20 µm, Dismic-25JP, Advantec, prior to injection to the HPLC system. The chromatographic peaks of sample solution were identified by comparing their retention time and UV spectra with those of standards. Quantitative analysis was carried out by integration of peak using external standard method.

The standard stock solutions of the four reference standards were dissolved in CHCl_3_/MeOH (7/3, *v/v*) and stored at 4 °C before use. The working solutions were prepared by serial dilution of stock solutions with CHCl_3_/MeOH (7/3, *v/v)*. Linear regression data were determined by calculation of integrated peak areas (*y*) of serial minimum concentrations *versus* each concentration (*x*, mg/mL).

### 3.5. Validation of the HPLC Methods

The HPLC analysis method was validated in terms of linearity, accuracy, precision, LOD, and LOQ following the guidance of the International Conference on Harmonization (ICH). Linearity was established by evaluating the value of *r*^2^ (correlation coefficient) in the calibration curve of ten serial concentrations. The precision test of the analysis method was examined by the intermediate evaluation method using measurements of the intra- and inter-day variability. Intra-day variability was determined by analyzing the sample solution during one of the study days (24 h), while inter-day variability was performed on four different days by injecting the sample solutions five times a day. Relative standard deviation (RSD) values were calculated for both retention time and peak area of five experiments. RSD was considered as a measure of precision. Recovery tests, meanwhile, were performed to evaluate accuracy in the sample solution spiked with each standard compound. Recovery rates were determined by calculating the mean recovery (%) of the standards from the spiked extract solutions *vs*. the non-spiked extract sample. LOD and LOQ were determined by the signal-to-noise ratio (S/N) method, in which an S/N ratio of 3 for LOD and an S/N ratio of 10 for LOQ were used.

### 3.6. Assay for Inhibition of TLR4 Activity

THP1-Blue cells, expressed from TLR1 to TLR10, were transfected with a reporter plasmid containing SEAP under the control of NF-κB and AP-1 inducible promoter. The activation of each TLR was determined by quantifying the secreted SEAP. Briefly, THP1-Blue-CD14 cells were seeded at a plating density of 2 × 10^5^ cells/well and the cells were treated with extract or compound for 1 h before incubating with LPS (1 μg/mL) for TLR4 activation. After 18 h incubation at 37 °C in a 5% CO_2_ atmosphere, 20 µL of cell suspension were added onto new 96-well plates and mixed with 200 µL of QUANTI-Blue colorimetric assay reagent (InvivoGen, San Diego, CA, USA) to detect SEAP. After incubation for 1 h at 37 °C for color development, quantitative reading was performed at 650 nm using a microplate reader.

## 4. Conclusions

In this study, an HPLC-UV method was developed for the quantitation of four compounds isolated from the ethanol extract of PAS: oleanolic acid (**1**), oleanolic acid acetate (**2**), stigmasterol (**3**) and β-sitosterol (**4**). To the best our knowledge, it was the first attempt to quantitate these four compounds in PAS, and the developed method was fully validated with good linearity (*r*^2^ > 0.999), accuracy (96.1%–104.4%), precision (<2.46%), LOD (<0.0014 mg/mL) and LOQ (<0.0043 mg/mL). This proposed procedure is easy to carry out and may provide an analytical basis of PAS. Also, TLR4 inhibitory effects of the extracts and isolated compounds were demonstrated without any cytotoxicity, and hereby was the leading component demonstrated as oleanolic acid acetate (**2**). Taken together, our findings clearly provide that PASE along with isolated compounds could be promising candidates for the inactivation of TLR4 signaling. Up to now, there have been no attempts to use PASE as a TLR4 inhibitor. We conclude that PASE might be serving as an antagonistic food against TLR4. However, further biological studies will be needed to establish its mechanism of action.

## References

[1] Itoh T., Umekawa H., Furuichi Y. (2005). Potential ability of hot water adzuki (*Vigna angularis*) extracts to inhibit the adhesion, invasion, and metastasis of murine B16 melanoma cells. Biosci. Biotechnol. Biochem..

[2] Itoh T., Itoh Y., Hibasami H., Katsuzaki H., Imai K., Furuichi Y., Komiya T. (2005). Vignoside, a novel new sesquiterpene glucoside obtained from a hot-water extract of adzuki beans (*Vigna angularis*). Nippon Shokuhin Kagaku Kougaku Kaishi.

[3] Yao Y., Cheng X., Wang L., Wang S., Ren G. (2011). A determination of potential α-glucosidase inhibitors from azuki beans (*Vigna angularis*). Int. J. Mol. Sci..

[4] Itoh T., Hori Y., Atsumi T., Toriizuka K., Nakamura T., Maeyama T., Ando M., Tsukamasa Y., Ida Y., Furuich Y. (2012). Hot water extract of adzuki beans (*Vigna angularis*) suppresses antigen-stimulated degranulation in rat basophilic leukemia RBL-2H3 cells and passive cutaneous anaphylaxis reaction in mice. Phytother. Res..

[5] Sato S., Mukai Y., Yamate J., Kato J., Kurasaki M., Hatai A., Sagai M. (2008). Effect of polyphenol-containing azuki beans (*Vigna angularis*) extract on blood pressure elevation and macrophage infiltration in the heart and kidney of spontaneously hypertensive rats. Clin. Exp. Pharmacol. Physiol..

[6] Oh H., Lee S.W., Yun B.R., Hwang B.S., Kim S.N., Park C.S., Jeoung S., Kim H., Lee W.S., Rho M. (2014). Vigna angularis inhibits IL-6-induced cellular signaling and ameliorates collagen-induced arthritis. Rheumatology.

[7] Yu T., Ahn H.M., Shen T., Yoon K., Jang H., Lee Y., Yang H., Kim J., Kim C., Han M. (2011). Anti-inflammatory activity of ethanol extract derived from Phaseouls angularis beans. J. Ethanopharmacol..

[8] Kim M.H., Jeoung S.H., Lee S.W., Kim H.K., Park C.S., Jeon B.H., Oh H.M., Rho M.C. (2012). Effect of Vigna angularis on toll-like receptor activation and pro-inflammatory cytokine production. Kor. J. Orient. Physiol. Pathol..

[9] Anwar M.A., Basith S., Choi S. (2013). Negative regulatory approaches to the attenuation of Toll-like receptor signaling. Exp. Mol. Med..

[10] Rho M.C., Lee W.S., Oh H.M., Kim Y.M., Ryu Y.B., Park S.J., Lee S.W., Cho K.O. (2014). Pharmaceutical Composition Containing Oleanolic Acid Acetate as an Active Ingredient for Preventing or Treating TLR- or IL-6-Mediated Diseases. U.S. Patent.

[11] Zacchigna M., Cateni F., Faudale M., Sosa S., Della Loggia R. (2009). Rapid HPLC analysis for quantitative determination of the two isomeric triterpenic acids, oleanolic acid and ursolic acid, in *Plantago Major*. Sci. Pharm..

[12] Unnati M., Shah S.M., Patel P.H., Hingorani L., adhav R.B. (2010). Development and validation of a simple isocratic HPLC method for simultaneous estimation of phytosterols in *Cissus quadrangularis*. Indian J. Pharm. Sci..

[13] Gohari A.R., Saeidnia S., Hadjiakhoondi A., Abdoullahi M., Nezafati M. (2009). Isolation and quantificative analysis of oleanolic acid from *Satureja mutica* Fisch. & C.A. Mey. J. Med. Plants Res..

[14] Werner S., Nebojsa S., Robert W., Robert S., Olaf K. (2003). Complete assignments of ^1^H and ^13^C-NMR resonances of oleanolic acid, 18α-oleanolic acid, ursolic acid and their 11-oxo derivatives. Magn. Reson. Chem..

[15] Naved T., Ansari S.H., Mukhtar H.M., Ali M. (2003). New triterpenic esters of oleanene-series from the flowers of *Calendula officinalis* Linn. Indian J. Chem..

[16] Kamboj A., Saluja A.K. (2011). Isolation of stigmasterol and β-sitosterol from petroleum ether extract of aerial parts of *Ageratum Conyzoides* (Asteraceae). Int. J. Pharm. Pharm. Sci..

